# Understanding parenting stress in autism spectrum disorder: a cross-sectional study exploring differences between mothers and fathers

**DOI:** 10.3389/fpsyg.2026.1947791

**Published:** 2026-09-18

**Authors:** Silvia Guerrera, Elisa Fucà, Martina Parisi, Stefano Vicari

**Affiliations:** 1 Child and Adolescent Neuropsychiatry Unit, Bambino Gesù Children's Hospital IRCCS, Rome, Italy; 2 Life Sciences and Public Health Department, Catholic University, Rome, Italy

**Keywords:** externalizing behaviors, fathers, internalizing behaviors, mothers, parenting

## Abstract

**Background:**

Parents of autistic children may experience high levels of stress. Prior research implicates child cognitive impairment, emotional and behavioral problems, sociodemographic factors, and autism symptoms as contributors; however, gender differences in parenting stress remain underexplored. This study compared parenting stress between mothers and fathers of autistic children and examined key predictors across stress domains.

**Methods:**

We retrospectively analyzed a database including data from 160 mother–father pairs of children aged 3–15 years who had received an ASD diagnosis. Parents completed the Parenting Stress Index (PSI) and measures of children's emotional/behavioral characteristics: We analyzed PSI domains: Parenting Distress (PD), Difficult Child (DC), and Parent–Child Dysfunctional Interaction (P-CDI). Analysis of covariance compared mothers and fathers. Multiple linear regressions identified predictors of each stress domain.

**Results:**

Mothers reported significantly higher scores than fathers only on PD. In mothers, child externalizing problems were the primary predictors of parenting stress, showing significant associations with PD, DC, and P-CDI. Restricted/repetitive behaviors were additionally associated with PD, and maternal education was associated with P-CDI. In fathers, no significant predictors emerged for PD. For other domains, child cognitive functioning, externalizing problems, and paternal education were associated with P-CDI, whereas externalizing problems were the sole significant predictor of DC.

**Discussion:**

These results suggests the relevance of considering mothers and fathers as separate yet complementary contributors within the family system, including when identifying determinants of parenting stress. Child externalizing problems are the principal vulnerability factor for both parents, underscoring the need for tailored support interventions for mothers and fathers.

## 1 Introduction

Over the past few decades, an expanding body of literature has emphasized the profound psychological burden experienced by families of autistic children. Autism spectrum disorder (ASD) is a neurodevelopmental condition characterized by persistent differences in social communication and the presence of restricted and repetitive behaviors (American Psychiatric Association, 2022). Global prevalence estimates range from 1 to 3.2% (Zeidan et al., 2022; Talantseva et al., 2023; Shaw et al., 2025). Autistic children present with heterogeneous profiles and varying levels of support needs, which differ according to developmental stage, cognitive functioning, co-occurring conditions, and the availability of appropriate services (Al-Beltagi, 2021). Autistic characteristics can frequently lead to relational difficulties within the family context, including recurrent communication challenges, difficulties in emotional expression, and atypical interaction patterns (Vivanti and Nuske, 2017). These daily relational challenges have consequences not only for the child but also demand continuous assistance and oversight, consequently placing a significant responsibility and pressure on caregivers, especially on parents (Li et al., 2022). Caregivers of autistic individuals consistently report intensified parenting stress (PS), and this burden frequently exceeds the distress felt by parents of neurotypical peers or children with alternative health conditions (Bouma and Schweitzer, 1990; Dumas et al., 1991; Kasari and Sigman, 1997; Sanders and Morgan, 1997; Davis and Carter, 2008; Rao and Beidel, 2009; Hayes and Watson, 2013; Giovagnoli et al., 2015). PS is commonly conceptualized as the psychological distress and discomfort arising from the demands and challenges associated with the parenting role (Deater-Deckard, 1998). In addition diminished efficacy in early intervention and poorer developmental outcomes—specifically regarding adaptive, educational, and cognitive skills—have been associated with high levels of PS (Robbins et al., 1991; Osborne et al., 2008). In recent years, considerable research efforts have focused on identifying the factors contributing to PS in families of autistic children (Abbeduto et al., 2004; Estes et al., 2009). Variables most frequently associated with parental stress include ASD symptom severity, cognitive impairment, emotional and behavioral difficulties, and socio-demographic characteristics (Schutte et al., 2018; Miranda et al., 2019).

Despite the growing literature on caregiver distress in families of autistic children, important gaps remain in understanding how parenting stress is experienced by mothers and fathers within the same family. Previous research has predominantly focused on mothers, whereas paternal stress has received comparatively less attention (Flippin and Crais, 2011; Giannotti et al., 2021; Johnson and Simpson, 2013; Rivard et al., 2014). Although studies including both parents have increased in recent years, findings regarding differences between maternal and paternal parenting stress remain inconsistent (Giannotti et al., 2021; Li et al., 2022; Perzolli et al., 2025; Rattaz et al., 2023). Moreover, less is known about whether child-related characteristics associated with parenting stress show similar or distinct patterns across mothers and fathers of autistic children (Davis and Carter, 2008). Considering both parental roles is particularly relevant because mothers and fathers may experience the demands associated with raising an autistic child differently, reflecting differences in caregiving responsibilities, employment, social support, and individual perceptions of parenting demands (Bristol et al., 1988; Hastings et al., 2005; Davis and Carter, 2008). Importantly, examining both parents within the same family provides an opportunity to characterize parental stress from complementary perspectives while also investigating whether the factors associated with stress are shared across parental roles or show parent-specific patterns (Grebe et al., 2022).

To address this literature gap, the present retrospective study investigated parenting stress in 160 mothers and 160 fathers of autistic children belonging to the same families. The primary objective was to examine differences in parenting stress between mothers and fathers across the three domains assessed by the Parenting Stress Index–Short Form: Parental Distress (PD), Parent–Child Dysfunctional Interaction (P-CDI), and Difficult Child (DC). The secondary objective was to investigate whether child-related characteristics—including cognitive functioning, ASD symptom severity, emotional and behavioral problems, and adaptive functioning—as well as parental sociodemographic characteristics, were associated with parenting stress in mothers and fathers, and whether the pattern of associations differed according to parental role. The present study aimed to extend previous research by examining maternal and paternal parenting stress within the same family context. We considered mothers and fathers as separate parental roles and examined both differences in parenting stress across PSI-SF domains and the patterns of child- and parent-related characteristics associated with stress within each role. This approach provided complementary information on maternal and paternal experiences of parenting stress while remaining distinct from a dyadic analysis of parental interdependence.

Two hypotheses have been formulated: (1) Mothers and fathers of autistic children would differ in parenting stress, with mothers expected to report higher levels of parental distress, as this dimension primarily reflects the parent's internal psychological states rather than demands directly related to the child; (2) child emotional and behavioral characteristics, particularly externalizing problems, would be associated with parenting stress in both mothers and fathers, although the strength and pattern of associations may differ between parental roles.

Considering the importance of individualizing interventions for autistic children and adolescents, there is an urgent need to develop targeted interventions that respond to specific family needs and to the distinct needs of mothers and fathers.

## 2 Materials and method

### 2.1 Procedure and participants

Data were extracted from a retrospectively developed database derived from an extensive review of the clinical records of patients referred to the Child and Adolescent Neuropsychiatry Unit of a tertiary-level Children's Hospital between 2020 and 2025 for neuropsychiatric assessment following a pediatrician's clinical suspicion of ASD or for clinical follow-ups after receiving ASD diagnosis. The participants included 160 mother–father pairs of children and adolescents who met the criteria for ASD. All mother–father pairs were living together with their child at the time of assessment. Specifically, some parents were married and cohabiting, whereas others were cohabiting without being married. Fathers were significantly older than mothers (mean age = 43.12 years, SD = 6.33 vs. 40.56 years, SD = 5.43; *p* < 0.001). Mothers had significantly higher educational attainment than fathers (mean years of education = 14.9, SD = 3.2 vs. 13.6, SD = 3.58; *p* < 0.001). Additionally, 38.8% of mothers were unemployed, compared with 6.9% of fathers (*p* < 0.01). Of the 160 children (2, 5–15 years; mean age: 6.57, SD = 2.61; 140 males, 20 females; mean non-verbal IQ: 73.84, SD = 20.77), 70 had co-occurring intellectual disability. All included participants had a diagnosis of ASD, with DSM-5-TR support levels ranging from Level 1 to Level 3. Regarding ASD symptomatology, the mean calibrated severity score (CSS) was 6 (SD = 1.5) for Social Affect (SA) and 7 (SD = 1.8) for Restricted and Repetitive Behaviors (RRBs). Routine assessment procedure included neuropsychiatric examination, cognitive and adaptive functioning evaluation, assessment of ASD symptoms and an emotional and behavioral evaluation.

### 2.2 Measures

#### 2.2.1 Parenting stress

To investigate parenting stress levels, the Parenting Stress Index-Short Form (PSI-SF) (Abidin, 1995; Guarino et al., 2008) was used. The PSI-SF is filled by parents and consists of 36 questions and each item is rated on a 5-point Likert scale from (1) strongly disagree to (5) strongly agree. It captures three domains—Parental Distress (PD), Parent–Child Dysfunctional Interaction (P-CDI), and Difficult Child (DC). The PD domain evaluates the extent of distress linked to personal challenges, including depression, conflicts with a partner, or limitations in life caused by the demands of parenting (e.g., “Since having this child, I have been unable to do new and different things”). The P-CDI domain reflects parental dissatisfaction with interactions with their children and the extent to which parents perceive their children as difficult or unacceptable (e.g., “My son rarely does things that make me feel good”). The DC domain assesses the child's self-regulatory capabilities as perceived by the parent (e.g., “My child seems to cry and get infatuated more often than most children”). The sum of all questions results in the Total Stress score. For this instrument, raw data were considered for the analyses. Data from the Italian validation test showed good psychometric properties (Cronbach's alpha ranging from 0.78 and 0.93). The instrument has been translated into several languages and has been frequently used in ASD research (Leonardi et al., 2021; Scibelli et al., 2021; Papanikolaou et al., 2022; Guerrera et al., 2025). In the present study, the PSI-SF was administered separately to both mothers and fathers. Thus, maternal and paternal scores were calculated independently for each PSI-SF domain and for Total Stress.

#### 2.2.2 Autism symptoms

The ASD diagnosis was determined according to DSM-5 criteria and subsequently validated through the use of the “gold-standard” tools for evaluating ASD symptoms, specifically the Autism Diagnostic Observation Schedule, Second Edition (ADOS-2) (Lord et al., 2012), and the Autism Diagnostic Interview-Revised (ADI-R; Lord et al., 1994). The ADOS-2 is a semi-structured direct assessment of communication, social interaction, and play or imaginative use of materials for individuals with a suspected diagnosis of ASD. The ADOS-2 consists of five modules designed for children and adults with different levels of language, ranging from non-verbal to verbal; it was administered and scored by licensed clinicians. Total score combines symptoms from the Social Affect (SA) and Restricted and Repetitive Behaviors (RRBs) domains. In the analyses, the CSS were considered for the ADOS-2. The ADI-R is a standardized, semi-structured interview during which caregivers report information about an individual suspected of having an ASD. The instrument generates algorithm scores for each of the three subdomains of autistic symptoms: qualitative impairments in reciprocal social behavior; qualitative abnormalities in communication and restricted range of interests and/or stereotypic behaviors.

#### 2.2.3 Cognitive assessment

Cognitive development was assessed by the Leiter International Performance Scale −3rd Edition—Leiter-3 (Roid et al., 2013)—which provides a non-verbal measure of intelligence. The Leiter-3 test is entirely non-verbal, including instructions, which are conveyed through non-verbal means. It consists of four subtests, namely: Figure Ground, where participants must identify a shape embedded within a complex figure; Form Completion, which requires participants to determine the form resulting from the combination of elements; Classifications and Analogies, where participants must classify forms and to reason through object matrices; and Sequential Order, which requires participants to discover the rule governing a series of forms and coherently add additional elements.

#### 2.2.4 Behavioral and psychological screening

Child Behavior Checklist (CBCL). Behavioral and psychological screening was performed by means of the parent-report questionnaire CBCL (Achenbach and Rescorla, 2007). For pre-schoolers, we used the CBCL for ages 1.5–5, which consists of 100 problem items. The instrument generates seven syndrome scales and five DSM-oriented scale profiles, consistent with the diagnostic categories of DSM-IV-TR and DSM-5. This version includes seven syndrome scales: Emotionally Reactive, Anxious/Depressed, Somatic Complaints, Withdrawn, Sleep Problems, Attention Problems, and Aggressive Behavior. The summary profile contains the Internalizing, Externalizing, and Total Problems scales. Finally, there are five DSM-oriented scale profiles, consistent with the diagnostic categories of DSM-IV-TR and DSM-5 (Affective problems, Anxiety problems, Pervasive Developmental Problems, Attention Deficit/Hyperactivity Problems—ADHD, and Oppositional Defiant Problems). For participants aged 6–18 years, we used the CBCL 6–18, which is composed of eight Empirically Based Syndromes Scales (Anxious/Depressed, Withdrawn/Depressed, Somatic Complaints, Social Problems, Thought Problems, Attention Problems, Rule-Breaking Behavior and Aggressive Behavior), three general domains (Total, Internalizing and Externalizing problems), and six Diagnostic and Statistical Manual of Mental Disorders (DSM)-oriented scales (Affective Problems, Anxiety Problems, Somatic Problems, Attention Deficit/Hyperactivity Problems, Oppositional Defiant Problems and Conduct Problems). In the current study, we considered the DSM-Oriented scales overlapping in the two versions of the instrument (i.e., versions for ages 1.5–5 and 6–18 years). Internal consistency and test-retest reliability of the Empirically Based Syndromes Scales were found to be good, with Cronbach Alphas ranging from 0.78 to 0.94 (Achenbach et al., 2003). Clinical Cut-off Description: T-Scores: 65–69 (Borderline) and 70+ (Clinical) are generated for narrow band scales. The CBCL was completed jointly by both parents. Therefore, a single shared parental report was available for each child.

#### 2.2.5 Adaptive skills

The Adaptive Behavior Assessment System II (ABAS-II) (Oakland, 2008) was used to investigate adaptive skills. The ABAS-II comprises five record forms: Parent/Primary Caregiver (for children ages 0–5), Teacher/Daycare Provider (for children ages 2–5), Parent (for children ages 5–21), Teacher (for children ages 5–21), and Adult (ages 16–89). The examiner gives selected forms to respondents such as parents, primary caregivers, teachers, daycare workers, counselors, who are familiar with the daily living circumstances of the individual. ABAS-II 0–5 and 5–21 yields three specific domain composite scores: Conceptual (communication, preschool/school skills, self-control), Social (play/leisure, socialization) and Practical (self-care, home/school life, use of the environment, health and safety, work) and an overall General Adaptive Composite. Added to these is the Motor skills area, which is limited to the assessment of children from 0 to 5 years old. The composite and domain scores are standard scores with a norm-referenced mean of 100 and standard deviation of 15. The Italian adaptation of the test showed good psychometric properties (Cronbach's alpha ranging from 0.92 and 0.99).

### 2.3 Statistical analyses

Descriptive statistics were calculated to characterize the demographic and clinical characteristics of the children and their parents, including means and standard deviations for continuous variables and frequencies and percentages for categorical variables. Child-related clinical characteristics included age, sex, non-verbal IQ, cognitive impairment, and ASD symptomatology as assessed by the ADOS-2. Parental characteristics included age, years of education, and employment status. To examine differences in parenting stress between mothers and fathers, four analyses of covariance (ANCOVAs) were conducted, with parental role (mother vs. father) as the between-subjects factor and parental age and years of education entered as covariates. The dependent variables were the three PSI-SF domains—PD, P-CDI, and DC. Separate multiple linear regression models were conducted for mothers and fathers to examine the associations of child- and parent-related characteristics with each PSI-SF domain. Separate multiple linear regression models were conducted for mothers and fathers. All prespecified predictors were entered simultaneously into each model using the enter method in SPSS (International Business Machines Corporation, United States); no stepwise or other data-driven variable selection procedure was applied. Predictor selection was based on their theoretical and clinical relevance to parenting stress and included child cognitive, autism-related, behavioral, adaptive, and demographic characteristics. The same set of child-related variables was entered into the maternal and paternal models, including non-verbal IQ, child age, ADOS-2 CSS for SA and RRBs, CBCL Internalizing and Externalizing Problems, and ABAS-II Conceptual, Social, and Practical domain scores. Maternal education was additionally included in the maternal models, whereas paternal education was included in the paternal models. CBCL variables represented a shared parental report and were therefore identical across the maternal and paternal regression models. A *p*-value ≤ 0.05 was considered statistically significant.

## 3 Results

### 3.1 Differences in parenting stress levels between fathers and mothers

In order to investigate differences in the levels of PS between mothers and fathers of autistic children, four ANCOVAs were conducted with parental role (mother vs. father) as the between-subjects factor, controlling for parental age and years of education. Regarding the PD subscale, a significant main effect of parental role emerged, with mothers reporting significantly higher scores than fathers, F (1, 316) = 5.131, *p* = 0.024, ηp^2^ = 0.016, after adjusting for the covariates. Regarding the P-CDI subscale, no significant differences emerged between mothers and fathers, F (1, 316) = 0.581, *p* = 0.446, ηp^2^ = 0.002. In relation to the DC subscale, mothers reported higher adjusted mean scores than fathers; however, this difference did not reach statistical significance, F (1, 316) = 2.048, *p* = 0.153, ηp^2^ = 0.006. Finally, in relation to the Total Stress, we did not observe main effect F (1, 316) = 3.147, *p* = 0.077, ηp^2^ = 0.01. Descriptive statistics are reported in Table 1.

**Table 1:** Parenting stress index- short form (PSI-SF) scores in mothers and fathers: means, standard deviations, and between-group comparisons.

PSI-SF domain	Mothers (*n* = 160)	Fathers (*n* = 160)	*p*
PD	27.68 ± 9.43	25.13 ± 8.91	0.024^*^
P-CDI	24.44 ± 7.04	24.63 ± 6.83	0.446
DC	31.16 ± 10.49	29.48 ± 9.56	0.153
Total stress	82.79 ± 23.03	77.69 ± 21.63	0.077

^*^P value ≤ 0.05; PD, parental distress; P-CDI, parent–child dysfunctional interaction; DC, difficult child.

### 3.2 Variables predicting parenting stress in mothers

Linear regression analysis revealed that some parental and child's features significantly predicted maternal PS levels for all the domains captured by the PSI-SF. Specifically, RRBs and child's externalizing behaviors significantly predicted parental distress (PD scale), with the model accounting for the 21.4% of variance (adjusted R^2^; *p* < 0.01), with child's externalizing behaviors having the highest predictive value. On the other side, child's externalizing behaviors and maternal education significantly predicted the perception of parent-child dysfunctional interaction (P-CDI scale), with the model accounting for the 34.2% of variance (adjusted R^2^; *p* < 0.01). Finally, only child's externalizing behaviors significantly predicted the perception of child behavioral difficulties (DC scale), with the model accounting for the 47.8% of variance (adjusted R^2^; *p* < 0.01). Tables 2–4 summarize the results.

**Table 2:** Linear regression predicting maternal parental distress (PSI–SF parental distress) from child cognitive ability (IQ) and age, ASD symptom severity (ADOS-2), emotional and behavioral problems (CBCL), adaptive functioning (ABAS-II), and maternal sociodemographic variables.

PARENT: MOTHER						
Outcome variable	Predictor	*B*	SE	*p*	*R^2^*	Adjusted *R^2^*
PSI-SF—Parental distress	IQ	−0.112	0.062	0.073	0.292	0.214
	ADOS-2 RRBs	−1.171	0.509	0.024		
	ADOS-2 social affect	0.101	0.599	0.866		
	CBCL internalizing problems	0.023	0.109	0.834		
	CBCL externalizing problems	0.421	0.105	< 0.001		
	Parental education	0.249	0.257	0.336		
	Child age	0.096	0.345	0.781		
	ABAS-II conceptual domain	−0.002	0.080	0.976		
	ABAS-II social domain	−0.044	0.100	0.663		
	ABAS-II practical domain	−0.005	0.076	0.950		

IQ, Intelligence quotient; RRBs, restricted and repetitive behaviors; ADOS-2, Autism Diagnostic Observation Schedule −2^nd^ edition; CBCL, Child Behavior Checklist; ABAS, Adaptive Behavior Assessment System.

**Table 3:** Linear regression predicting maternal parent–child dysfunctional interaction (PSI–parent–child dysfunctional interaction) from child cognitive ability (IQ) and age, ASD symptom severity (ADOS-2), emotional and behavioral problems (CBCL), adaptive functioning (ABAS-II), and maternal sociodemographic variables.

PARENT: MOTHER						
Outcome variable	Predictor	*B*	SE	*p*	*R^2^*	Adjusted *R^2^*
PSI-SF—parent-child dysfunctional interaction	IQ	−0.066	0.044	0.141	0.408	0.342
	ADOS-2 RRBs	−0.209	0.363	0.567		
	ADOS-2 social affect	−0.414	0.427	0.335		
	CBCL internalizing problems	0.123	0.078	0.118		
	CBCL externalizing problems	0.262	0.075	0.001		
	Parental education	0.466	0.183	0.013		
	Age	0.441	0.246	0.077		
	ABAS-II conceptual domain	−0.004	0.057	0.948		
	ABAS-II social domain	−0.095	0.071	0.183		
	ABAS-II practical domain	−0.013	0.054	0.811		

IQ, Intelligence quotient; RRBs, restricted and repetitive behaviors; ADOS-2, Autism Diagnostic Observation Schedule −2^nd^ edition; CBCL, Child Behavior Checklist; ABAS, Adaptive Behavior Assessment System.

**Table 4:** Linear regression predicting maternal perception of difficult child (PSI–difficult child) from child cognitive ability (IQ) and age, ASD symptom severity (ADOS-2), emotional and behavioral problems (CBCL), adaptive functioning (ABAS-II), and maternal sociodemographic variables.

PARENT: MOTHER						
Outcome variable	Predictor	*B*	SE	*p*	*R^2^*	Adjusted *R^2^*
PSI PSI-SF—Difficult child	IQ	−0.012	0.060	0.839	0.529	0.478
	ADOS-2 RRBs	−0.281	0.490	0.567		
	ADOS-2 social affect	−0.143	0.576	0.805		
	CBCL internalizing problems	0.104	0.105	0.322		
	CBCL externalizing problems	0.684	0.101	< 0.001		
	Parental education	−0.208	0.247	0.403		
	Age	−0.013	0.332	0.970		
	ABAS-II conceptual domain	0.020	0.077	0.797		
	ABAS-II social domain	0.047	0.096	0.624		
	ABAS-II practical domain	−0.107	0.073	0.149		

IQ, Intelligence quotient; RRBs, restricted and repetitive behaviors; ADOS-2, Autism Diagnostic Observation Schedule −2^nd^ edition; CBCL, Child Behavior Checklist; ABAS, Adaptive Behavior Assessment System.

### 3.3 Variables predicting parenting stress in fathers

Linear regression analyses revealed that child-related characteristics significantly predicted paternal PS in two PSI-SF domains. Specifically, the model predicting paternal parental distress was not significant (*p* = 0.18). In contrast, child IQ, externalizing behaviors, and paternal educational level significantly predicted perceptions of parent–child dysfunctional interaction (P-CDI scale), with the model explaining 19.3% of the variance (adjusted R^2^, *p* = 0.01). Consistent with the findings observed for mothers, child externalizing behaviors emerged as the only significant predictor of paternal perceptions of child behavioral difficulties (DC scale), and the model accounted for 18.9% of the variance (adjusted R^2^, *p* = 0.01). Tables 5–7 summarize the results.

**Table 5:** Linear regression predicting paternal parental distress (PSI–parental distress) from child cognitive ability (IQ), ASD symptom severity (ADOS-2), emotional and behavioral problems (CBCL), adaptive functioning (ABAS-II), and paternal sociodemographic variables.

PARENT: FATHER						
Outcome variable	Predictor	*B*	SE	*p*	*R^2^*	Adjusted *R^2^*
PSI-SF—Parental distress	IQ	−0.052	0.069	0.451	0.133	0.040
	ADOS-2 RRBs	−0.145	0.562	0.797		
	ADOS-2 social affect	−0.173	0.674	0.798		
	CBCL internalizing problems	0.125	0.126	0.322		
	CBCL externalizing problems	0.188	0.119	0.117		
	Parental education	0.509	0.274	0.066		
	Age	0.080	0.394	0.839		
	ABAS-II conceptual domain	−0.060	0.093	0.522		
	ABAS-II social domain	−0.027	0.113	0.810		
	ABAS-II practical domain	0.017	0.085	0.839		

IQ, Intelligence quotient; RRBs, restricted and repetitive behaviors; ADOS-2, Autism Diagnostic Observation Schedule −2^nd^ edition; CBCL, Child Behavior Checklist; ABAS, Adaptive Behavior Assessment System.

**Table 6:** Linear regression predicting paternal parent–child dysfunctional interaction (PSI–parent–child dysfunctional interaction) from child cognitive ability (IQ) and age, ASD symptom severity (ADOS-2), emotional and behavioral problems (CBCL), adaptive functioning (ABAS-II), and paternal sociodemographic variables.

PARENT: FATHER						
Outcome variable	Predictor	*B*	SE	*p*	*R^2^*	Adjusted *R^2^*
PSI-SF—Parent-child dysfunctional interaction	IQ	−0.110	0.045	0.017	0.272	0.193
	ADOS-2 RRBs	0.457	0.367	0.216		
	ADOS-2 social affect	−0.117	0.440	0.792		
	CBCL internalizing problems	0.077	0.082	0.350		
	CBCL externalizing problems	0.193	0.078	0.015		
	Parental education	0.384	0.179	0.035		
	Age	0.062	0.257	0.810		
	ABAS-II conceptual domain	0.008	0.061	0.899		
	ABAS-II social domain	0.022	0.074	0.771		
	ABAS-II practical domain	−0.054	0.056	0.337		

IQ, Intelligence quotient; RRBs, restricted and repetitive behaviors; ADOS-2, Autism Diagnostic Observation Schedule −2^nd^ edition; CBCL, Child Behavior Checklist; ABAS, Adaptive Behavior Assessment System.

**Table 7:** Linear regression predicting paternal perception of difficult child (PSI–difficult child) from child cognitive ability (IQ) and age, ASD symptom severity (ADOS-2), emotional and behavioral problems (CBCL), adaptive functioning (ABAS-II), and paternal sociodemographic variables.

PARENT: FATHER						
Outcome variable	Predictor	*B*	SE	*p*	*R^2^*	Adjusted *R^2^*
PSI-SF—Difficult child	IQ	−0.037	0.060	0.537	0.268	0.189
	ADOS-2 RRBs	−0.235	0.490	0.632		
	ADOS-2 social affect	−0.048	0.588	0.936		
	CBCL internalizing problems	0.136	0.110	0.220		
	CBCL externalizing problems	0.393	0.104	< 0.001		
	Parental education	0.321	0.239	0.182		
	Age	0.112	0.344	0.745		
	ABAS-II conceptual domain	−0.050	0.081	0.536		
	ABAS-II social domain	0.033	0.099	0.743		
	ABAS-II practical domain	0.040	0.074	0.593		

IQ, Intelligence quotient; RRBs, restricted and repetitive behaviors; ADOS-2, Autism Diagnostic Observation Schedule −2^nd^ edition; CBCL, Child Behavior Checklist; ABAS, Adaptive Behavior Assessment System.

## 4 Discussion

### 4.1 Parenting stress differences

The present study examined PS in a large sample of parental couples of autistic youths, with the primary aim of investigating potential differences in perceived parenting stress between mothers and fathers. The first finding of this study revealed significant differences between mothers and fathers only for the PD subscale, with maternal cohorts exhibiting markedly elevated distress relative to paternal caregivers. Our findings appear to be consistent with some previous literature. Notwithstanding a historical paucity of research evaluating paternal cohorts (Flippin and Crais, 2011), fathers appear to sustain a caregiver burden comparable to that of mothers, demonstrating a similar vulnerability to the daily demands of raising an autistic child. In line with this, several investigations have detected no statistically significant gender discrepancies regarding PS (Ozturk et al., 2014; Ramírez et al., 2026). Non-etheless, empirical findings on gender-related variations remain inconsistent, as a substantial body of literature conversely links maternal status to higher levels of clinically significant distress (Hastings and Brown, 2002; Herring et al., 2006). Overall, studies over time have shown increasingly comparable stress levels between mothers and fathers (Davis and Carter, 2008). This growing convergence in stress profiles may stem from a generational shift, with contemporary fathers demonstrating higher levels of engagement in daily childcare and symptom management compared to their predecessors (Pleck and Masciadrelli, 2004). Conversely, recent evidence investigating clinical and sociodemographic correlates among Australian parents of preschool-aged autistic children indicated that mothers still endure higher mean total stress, driven predominantly by elevated scores within the parental distress domain (John et al., 2026). Aligning partially with our outcomes, the maternal discrepancies observed on the PD dimension could be explained by women's broader susceptibility to stressful circumstances and their tendency to evaluate domestic and family-related events with greater psychological severity than men (Ferreira et al., 2021). The PD scale captures both the positive and negative emotional dimensions of caregiving, encompassing components such as depressive symptoms, perceived restrictions, conflict, social support, and sense of competence. Consequently, intergroup variations observed within this subscale likely emerge because this dimension heavily captures caregivers' intrinsic psychological traits and internal dispositions, remaining relatively detached from child-specific characteristics (Giannotti et al., 2023). An additional explanation, linked to the sociodemographic profile of our sample, relates to maternal employment dynamics; despite possessing advanced academic qualifications, mothers in our cohort report lower rates of professional employment compared to their partners. This socioeconomic imbalance potentially intensifies the subjective appraisal of domestic stressors, leading to elevated maternal PD scores—a phenomenon documented across alternative neurodevelopmental contexts (Fucà et al., 2025). In summary, the substantial divergence in parental distress findings across the literature may arise from methodological heterogeneities. These include variations in participant demographics—such as familial background, sample size, chronological age of the children, and specific stages of the family life cycle—alongside discrepancies in psychometric instruments and the non-uniform clinical thresholds utilized to establish stress severity (Rivard et al., 2014).

### 4.2 Predictors of parenting stress

With regard to maternal and paternal distress in relation to child characteristics, our second main finding was that repetitive and externalizing behaviors significantly predicted maternal distress, with externalizing behaviors showing a stronger predictive effect. This finding aligns with previous studies demonstrating that emotional and behavioral problems in children and in particular child externalizing behaviors have been more consistently associated with increased parental stress: the psychological distress of caregivers is often compounded by the constant supervision required to manage these highly prominent and disruptive behaviors (Perzolli et al., 2025). Moreover, the observation that externalizing behaviors predicted PD scores among mothers, but not fathers, may be linked to a greater perceived impact of these behaviors on maternal mental health. Prior studies have shown that the tendency of mothers to overestimate their child's behavioral issues, or to experience them as substantially more taxing, may be driven by their own elevated baseline of psychological distress and mental health challenges relative to fathers (Schnabel et al., 2020; Rattaz et al., 2023). Externalizing behaviors emerged as significant predictors of maternal stress across both the P-CDI and DC subscales. This finding is conceptually plausible, as externalizing behaviors may negatively influence the perceived quality of parent–child interactions and, as previously reported by Huang and colleagues, the Difficult Child dimension of PS is closely associated with children's behavioral problems (Huang et al., 2014). Taken together, these results indicate that externalizing behaviors deeply influences how caregivers perceive both their child's disposition and the quality of their mutual interactions, framing them as increasingly complex (Scibelli et al., 2021). Regarding repetitive behaviors, in line with the literature—which generally indicates that the association between maternal stress and ASD symptom severity is weak and predominantly driven by restricted and repetitive behaviors—our findings are consistent with this pattern (Giannotti et al., 2021; John et al., 2026), although this association with the PD subscale has not always been observed (Scibelli et al., 2021). Such discrepancies are elucidated by prior models focusing on parental strain (McStay et al., 2014; Mello et al., 2022), which demonstrate that once externalizing tendencies are statistically controlled, the unique variance explained by core ASD severity diminishes significantly, being heavily suppressed by the former. Finally, in line with previous literature investigating the relationship between sociodemographic factors and PS, our outcomes indicate that elevated paternal and maternal education might act as a vulnerability marker, correlating with intensified stress profiles and reduced psychological wellbeing. Rather than acting as a buffer, higher academic backgrounds may amplify parental vigilance or cognitive sensitivity toward relational challenges within the household, a trend that manifests uniformly across both genders (Falk et al., 2014; Hodgetts et al., 2017). Alternative explanations should also be considered. Higher stress among more highly educated parents may reflect greater awareness of ASD, its developmental course, available interventions, and prognostic outcomes, leading to increased concern about their child's future. In addition, these parents may hold higher expectations for their child's developmental trajectory, making discrepancies between expectations and reality more psychologically distressing. Greater occupational responsibilities and associated cognitive demands may further contribute to elevated levels of parental stress. From a clinical perspective, these findings suggest that highly educated parents should not necessarily be considered at lower risk of parenting stress simply because they may have greater access to information or resources. Clinicians may benefit from exploring parents' expectations, perceptions of their child's developmental trajectory, and the emotional impact of extensive information-seeking, rather than assuming that greater knowledge is inherently protective. Providing individualized psychoeducation, realistic developmental expectations, and opportunities to discuss uncertainty may help parents integrate information about ASD without increasing perceived pressure or responsibility. Importantly, these considerations should be viewed as hypotheses for clinical attention rather than as direct implications causally established by the present study. With regard to paternal PS, none of the examined variables significantly contributed to the PD dimension. However, in line with findings observed in mothers, child externalizing behaviors and paternal educational level emerged as significant predictors of stress in the P-CDI domain, while child externalizing behaviors were again identified as a significant predictor of paternal stress in the DC subscale. This pattern is consistent with previous evidence suggesting that fathers' psychological wellbeing may be more strongly influenced by their children's behavioral difficulties than by the severity of ASD symptomatology (McStay et al., 2014; Li et al., 2022; John et al., 2026). Conversely, when interpreted within a broader framework, these findings suggest that child externalizing behaviors represent a relevant predictor of stress for both maternal and paternal stress. From a broader theoretical perspective, these findings may be consistent with transactional models in which child behavioral difficulties and parental stress potentially influence one another over time. Therefore, addressing these reciprocal processes through a bidirectional approach may facilitate the development of family-centered interventions that simultaneously target child behavioral difficulties and parental wellbeing. However, given the cross-sectional and retrospective nature of the present study, the directionality of these associations cannot be established, and the possibility that parental stress contributes to subsequent increases in externalizing behaviors remains a theoretical interpretation rather than an empirical finding of the current study. Longitudinal studies are needed to determine whether reciprocal processes between child behavior and parental wellbeing emerge over time.

Regarding the role of intellectual functioning as a predictor of paternal stress, specifically within the P-CDI subscale, to our knowledge, no previous studies on ASD population investigating gender differences in PS have reported comparable findings. Existing research has heavily centered on the contribution of clinical features (including IQ, behavioral difficulties, and functional impairment) to parental stress. However, literature consistently affirms that externalizing behavioral issues generate a far more potent impact on parental distress than the clinical diagnostic severity *per se*, independent of whether the caregiver is the mother or the father (Willoughby and Glidden, 1995; Essex et al., 1999; Hastings et al., 2005; Giovagnoli et al., 2015). A recent Italian study examining parental stress and parent-reported perceptions of children's behaviors and symptom severity found no significant associations between PS and children's cognitive functioning (Perzolli et al., 2025). Conversely, Scibelli et al. (2021) reported that cognitive functioning was the only child-related variable associated with PS across all subscales. These discrepant findings should be interpreted considering the substantial methodological heterogeneity characterizing the existing literature, particularly regarding sample size, participants' age range, outcome measures, and assessment tools employed. Moreover, our findings should be considered in light of the relatively low mean cognitive functioning observed in the present sample.

### 4.3 Limitations and conclusions

Several limitations of this study should be acknowledged. First, the retrospective design prevents any causal interpretation of the observed associations. Future longitudinal studies are warranted to clarify the temporal dynamics and potential mechanisms underlying these relationships. Second, children's internalizing and externalizing behaviors were assessed through parent-report measures, which may not fully capture objective aspects of child functioning or parental characteristics; moreover, children's internalizing and externalizing behaviors were assessed using a shared parental report. Although this approach provided a joint parental perspective, separate maternal and paternal reports were not available, preventing the examination of potential differences in parents' perceptions of their child's behavior and their possible contribution to parenting stress. Third, maternal and paternal mental health variables were not included, which could have provided further insight into the factors contributing to parental stress. Fourth, the wide age range of the children included in our sample represents an additional limitation. Although this broad developmental span extends the scope of the study by capturing different stages of child development, parenting demands, environmental and social challenges, and school-related transitions may vary substantially across these developmental periods. Consequently, this heterogeneity may limit the identification of age-specific patterns, including potential periods of increased PS associated with specific developmental stages. Fifth, the study did not include a measure of parental caregiving involvement. Therefore, the observed differences between mothers and fathers may partly reflect differences in their degree of daily hands-on involvement in childcare rather than biological sex alone. Future studies should assess parental involvement and caregiving responsibilities directly to disentangle the contribution of caregiving roles from that of parent sex in explaining differences in PS.

Despite these limitations, the present study also presents several strengths. First, it is among the relatively few investigations to examine PS in both mothers and fathers, reflecting the growing recognition of the importance of considering both caregivers within the family system. Second, the relatively large sample size, involving a substantial number of families, increases the reliability and generalizability of the findings. Third, this study contributes to a more comprehensive understanding of PS by examining not only overall stress levels but also specific dimensions reflecting different aspects of the parent–child relationship.

Overall, the findings of the present study highlight the importance of considering the broader family context when examining the interplay between child characteristics and parental wellbeing in families of autistic children. The distinct experiences reported by mothers and fathers suggest the need to consider both caregivers as separate yet complementary contributors within the family system, each providing unique insights into overall family functioning. In this context, fathers' perspectives may offer valuable information regarding family dynamics and should therefore be more consistently acknowledged and incorporated into both future research and clinical practice. Furthermore, the observed association between child externalizing behaviors and parental distress suggests that interventions targeting behavioral difficulties may benefit from the active involvement of both parents and from the promotion of shared coping strategies. A coordinated parental approach to managing challenging behaviors may not only contribute to reducing perceived parenting burden but also support more adaptive family functioning. Taken together, these findings reinforce the importance of implementing individualized and inclusive interventions aimed at addressing the needs of the entire family system.

## Data Availability

The raw data supporting the conclusions of this article will be made available by the authors, without undue reservation.
